# Elimination of laboratory ozone leads to a dramatic improvement in the reproducibility of microarray gene expression measurements

**DOI:** 10.1186/1472-6750-7-8

**Published:** 2007-02-12

**Authors:** William S Branham, Cathy D Melvin, Tao Han, Varsha G Desai, Carrie L Moland, Adam T Scully, James C Fuscoe

**Affiliations:** 1 Center for Functional Genomics, Division of Systems Toxicology, National Center for Toxicological Research, U. S. Food and Drug Administration, 3900 NCTR Road, HFT-130, Jefferson, Arkansas 72079, USA; 2 Arkansas Regional Laboratory, Office of Regulatory Affairs, U. S. Food and Drug Administration, 3900 NCTR Road, SWR-570, Jefferson, Arkansas 72079, USA; 3 Facilities Design and Construction, National Center for Toxicological Research, U. S. Food and Drug Administration, 3900 NCTR Road, HFT-130, Jefferson, Arkansas 72079, USA

## Abstract

**Background:**

Environmental ozone can rapidly degrade cyanine 5 (Cy5), a fluorescent dye commonly used in microarray gene expression studies. Cyanine 3 (Cy3) is much less affected by atmospheric ozone. Degradation of the Cy5 signal relative to the Cy3 signal in 2-color microarrays will adversely reduce the Cy5/Cy3 ratio resulting in unreliable microarray data.

**Results:**

Ozone in central Arkansas typically ranges between ~22 ppb to ~46 ppb and can be as high as 60–100 ppb depending upon season, meteorological conditions, and time of day. These levels of ozone are common in many areas of the country during the summer. A carbon filter was installed in the laboratory air handling system to reduce ozone levels in the microarray laboratory. In addition, the airflow was balanced to prevent non-filtered air from entering the laboratory. These modifications reduced the ozone within the microarray laboratory to ~2–4 ppb. Data presented here document reductions in Cy5 signal on both in-house produced microarrays and commercial microarrays as a result of exposure to unfiltered air. Comparisons of identically hybridized microarrays exposed to either carbon-filtered or unfiltered air demonstrated the protective effect of carbon-filtration on microarray data as indicated by Cy5 and Cy3 intensities. LOWESS normalization of the data was not able to completely overcome the effect of ozone-induced reduction of Cy5 signal. Experiments were also conducted to examine the effects of high humidity on microarray quality. Modest, but significant, increases in Cy5 and Cy3 signal intensities were observed after 2 or 4 hours at 98–99% humidity compared to 42% humidity.

**Conclusion:**

Simple installation of carbon filters in the laboratory air handling system resulted in low and consistent ozone levels. This allowed the accurate determination of gene expression by microarray using Cy5 and Cy3 fluorescent dyes.

## Background

To obtain valid results from microarray experiments, many factors affecting microarray technology must be recognized and controlled [1-3]. Microarray analysis of gene expression depends on the relative binding (hybridization) of cyanine dye-labeled cDNAs or cRNAs to DNA probes covalently attached to microscope slides. The quality of microarray data depends on many factors and among the most important are stable ratios of the bound Cy5 and Cy3 dyes. Thus, each dye must remain intact from the completion of the hybridization process through the duration of the scanning process. The cyanine dye Cy5 is subject to ozone (0_3_) oxidation resulting in a decrease in fluorescence intensity [4]. Because two-color microarray experiments depend on the ratio of the Cy5 and Cy3 signal intensities for relative gene expression measurements, specific, rapid, and uncontrolled degradation of the Cy5 dye would result in inaccurate gene expression/repression (Cy5/Cy3) ratios and erroneous interpretation of microarray data. Oxidation occurs primarily after the hybridization washing procedures have been completed and the microarray becomes exposed to air containing environmental ozone.

Ozone in the lower atmosphere is present at all times of the year and is generally higher during the summer months. Ozone is a principal component of smog so ozone levels are higher in urban and/or industrialized areas compared with rural areas. The mixture of hydrocarbons and nitrogen oxide from automobile exhaust and factory emissions combined with exposure to sunlight in the presence of little air movement leads to the generation of ozone in the lower atmosphere [5]. Cy5 oxidation can occur at ozone levels as low as 5–10 ppb and possibly even lower. Average monthly ozone levels measured in central Arkansas between 1 January 2005 and 30 June 2005 ranged between 16 ppb and 43 ppb. However individual hourly readings reached as high as 103 ppb during this interval [6]. In addition to seasonal changes, a diurnal variation of ozone levels occurs because more ozone is produced as more atmospheric pollutants are generated during daylight hours.

To overcome this widespread problem, a simple laboratory engineering solution was developed that resulted in low and consistent ozone levels throughout the entire laboratory. The data presented here demonstrate the effects of both ozone and humidity on microarray fluorescence measurements for both in-house fabricated arrays and those manufactured by Agilent Technologies (Palo Alto, CA, USA), and the dramatic improvement in fluorescent dye stability under the newly implemented carbon filtration system. We also provide several other simple practices that can reduce the detrimental impact of atmospheric ozone on microarray experiments.

## Results

**Figure 1:**
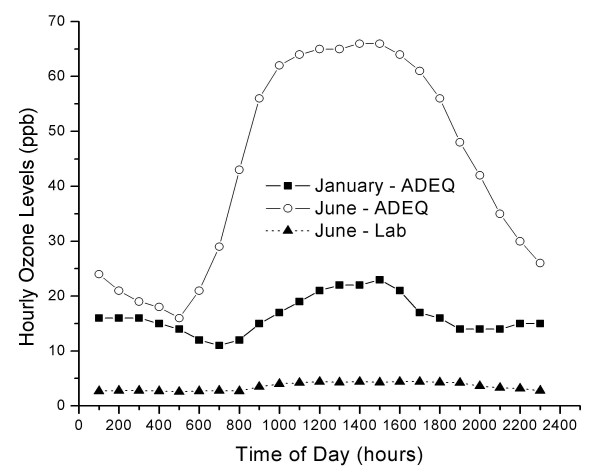
Diurnal ozone fluctuations. The means of hourly atmospheric ozone levels measured during January and June 2005 in central Arkansas by the ADEQ along with carbon-filtered laboratory air are shown. Peak ozone concentrations averaged 56–66 ppb between 0900 and 1800. Hourly ozone levels during this period commonly reached 60–90 ppb with the highest level reaching 103 ppb. By contrast, laboratory ozone levels varied between 2.6 and 4.4 ppb after installation of the carbon-filtered air supply.

An extensive database for air quality is maintained by the Arkansas Department of Environmental Quality (ADEQ) and was made available for this project. Figure 1 shows the average ozone concentrations during the months of January 2005 and June 2005, and illustrates the substantial seasonal differences in ozone levels in our region of central Arkansas. These data also show the diurnal fluctuation of the ozone level with the lowest level during the early morning hours and a peak between the hours of approximately 0900 and 1800. The average daily ground ozone level, in June 2005 ranged from 16 ppb to 66 ppb with individual hourly readings often in the 70–90 ppb range; a high of 103 ppb ozone was reached on June 28. Ozone readings within the microarray laboratory were similar to the environmental levels.

To reduce the laboratory ozone levels, a High-Efficiency Gas Adsorber (HEGA) carbon filter was installed in the laboratory air supply system (see Methods section for details). The airflow was also adjusted to keep the laboratory air pressure positive with respect to the hallway, thus preventing non-filtered air from entering the laboratory. Ozone readings taken within the carbon-filtered lab during June 2005 ranged from 2.6 ppb to 4.4 ppb (Figure 1). While filtration reduced the ozone levels to well below environmental ozone levels outside the lab, a slight diurnal variation is also seen within the lab. Two additional changes were also made in our scanning protocol. First, microarray hybridization experiments were scheduled so that washing and scanning were conducted early in the morning hours (0700–0900) when ozone levels are the lowest. Second, the microarray slides were scanned immediately after the final wash by spinning the slide dry for 10 seconds at 6,000 rpm using a micro-centrifuge equipped with a microscope slide adaptor and placing the microarray directly into the scanner. Fare et al. [4] demonstrated that degradation of the Cy5 signal by ozone is not significant as long as the slides remain in liquid.

**Figure 2:**
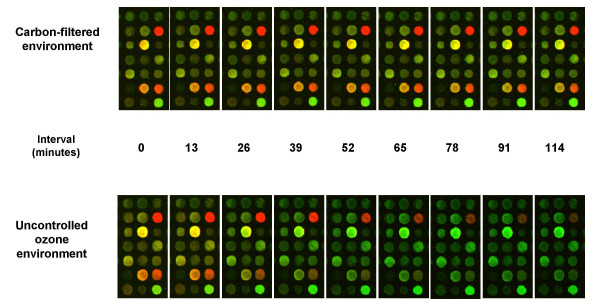
Reduction of Cy5 signal for in-house fabricated microarrays kept in a none-ozone controlled environment. These figures show a region of two 20 K mouse microarrays, selected because of their wide range of both Cy5 and Cy3 signals. The microarray images were from scans made during a 114 minute experiment interval. While the microarrays maintained in the carbon-filtered laboratory environment (ozone ~2–4 ppb) remained relatively unchanged with time (top images), reduction in Cy5 in the uncontrolled ozone environment (ozone ~25 ppb) causes the microarray image to have a predominantly green cast (bottom images).

The effects of ozone on an in-house fabricated microarray are shown in Figure 2. In this experiment, pairs of hybridized microarrays were first scanned in the carbon filtered laboratory. Following this, one of the microarrays was moved to a loosely covered slide box in the adjacent hallway where ozone was present at 25 ppb, while the other was kept in the carbon-filtered laboratory environment. The slides were scanned alternately in the carbon-filtered lab. Thus, the only time the slide was exposed to ozone was during the time it was in the hallway, which was for approximately 57 minutes during the 114 minute experiment. The reduction in Cy5 (red) signal in individual features (Cy5 dye excited at 635 nm) was dramatic across this time period. Similar reductions in Cy5 intensities were also observed in Agilent microarrays (images not shown).

**Figure 3:**
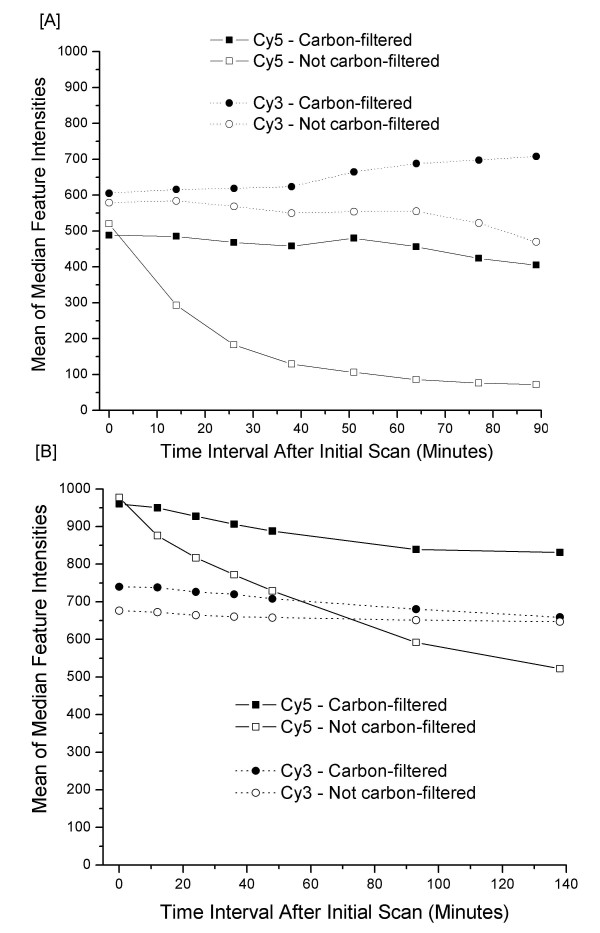
Dramatic improvement of Cy5 fluorescence stability as a result of ozone depletion. Pairs of microarrays printed in-house [A] or printed by Agilent Technologies [B] were hybridized and initially scanned immediately after washing in the carbon-filtered lab. One of each of the pairs of slides was then moved to an environment in which ozone was not removed by carbon filtration (ozone ~25 ppb for the in-house produced slide and ~10 ppb for the Agilent slide). The remaining slide remained in the ozone controlled microarray laboratory (ozone ~2–4 ppb). The slides were then alternately rescanned 6 times. The data show the rapid decline in Cy5 feature intensities as early as 13 minutes in the non-ozone controlled environment. It should be noted that the "ozone-exposed" microarray was not exposed to ozone while it was being scanned.

Several experiments were conducted to establish a time course for microarray degradation in a non-carbon-filtered atmosphere and to demonstrate the improvement due to the procedural and engineering modifications described above. Figure 3A shows median feature intensity data from microarrays printed in-house that were scanned immediately after washing in the carbon-filtered laboratory (ozone ~2–4 ppb). After scanning, one of the microarrays was moved to the adjacent hallway which does not have carbon-filtered supply air. The hallway was used as an ozone exposure environment to enable direct measurement of ozone in both locations and to move the microarray into the scanner as quickly as possible during the experiment. The microarrays were scanned alternately with approximately 12–14 minutes between individual slide scans. This was the minimum time to scan the microarray, save the image data to the computer, and change to the alternate microarray. One microarray slide never left the carbon-filtered laboratory environment, while the other slide was returned to the non-carbon-filtered hallway after each scan. During the first 13 minutes, the reduction of the Cy5 signal was approximately 44% in the non-carbon-filtered environment (ozone level was ~25 ppb) while the reduction was less than 1% in the carbon-filtered laboratory. By contrast there was no reduction in Cy3 intensity. After 39 minutes, Cy5 intensity was reduced by 6% in the microarray maintained in the carbon-filtered laboratory environment and by 75% in the microarray from the non-carbon-filtered environment. Cy3 intensity increased slightly in the carbon-filtered laboratory, but decreased by about 5% in the non-carbon-filtered hallway after 39 minutes. Such experiments were repeated two times with similar results. Thus, using these microarray scanning procedures and engineering modifications, ozone degradation of the Cy5 dye was minimized.

The question arose as to whether microarrays supplied by a commercial vendor would respond to ozone differently than those printed "in-house". To examine this, an ozone degradation experiment was conducted on two hybridized Agilent Technologies microarrays (Fig. 3B). These results show a rapid decline in the Cy5 signal that is very similar to that observed using microarrays printed in-house. Ozone levels in the carbon-filtered laboratory remained constant at about 2–4 ppb; ozone in the non-carbon-filtered hallway was approximately 10 ppb during the scanning duration.

To address the possibility that normalization might be used to correct for the uncontrolled degradation of the Cy5 intensity in a non-carbon-filtered laboratory environment, LOWESS normalization was applied to both the non-carbon-filtered and carbon-filtered data sets from the in-house printed arrays. Table 1 shows the Pearson correlation coefficients of spot intensities and Cy5/Cy3 ratios for the 8 successive scans of the slides when compared to the initial scan. Under conditions of low laboratory ozone (carbon-filtered), the un-normalized ratio correlations were very high, ranging from 0.99 at early scan times to 0.96 at the 91 minute scan. Normalization slightly improved the correlations at the 78 and 91 minute times. The correlation coefficients for the Cy3 and Cy5 intensity data were 0.99, even at the 91 minute scan time. Thus, consistent results were obtained whether the slides were scanned immediately or scanned 91 minutes later. When ozone is not controlled (non-carbon filtered), the correlations of the un-normalized ratio data declined dramatically with time. Normalization was able to improve the correlations, although not to the extent of data collected in the ozone controlled conditions. While the Cy3 intensity correlations remained high over time, the Cy5 intensity correlations decreased with time. Thus, normalization does not overcome the deleterious effects of ozone exposure.

The effect of high humidity alone on the stability of the cyanine dyes was also assessed. Twenty in-house fabricated microarrays containing 10,000 rat oligonucleotides were hybridized with Cy3- and Cy5-labeled rat cDNA. They were initially scanned in the carbon-filtered laboratory environment in which the relative humidity was maintained at 42%. Following this, 10 microarray slides were placed in a humidified chamber at a constant humidity of 98–99% and the remaining 10 slides were left in the carbon-filtered, 42% humidity laboratory environment. After 2 hours and 4 hours, 5 slides from the high humidity chamber and 5 slides kept under laboratory environment conditions were rescanned at the original PMT/power settings. The data indicated little change in the median feature intensities of either cyanine dye when the slides were maintained at 42% humidity for 2 hours [Cy5 mean % change ± SD = -0.2 ± 1.3%; Cy3 mean % change ± SD = 1.0 ± 2.1%] or four hours [Cy5 mean % change ± SD = -6.6 ± 3.3%; Cy3 mean % change ± SD = 2.2 ± 3.3%]. However, modest but significant increases in cyanine dye intensities were seen after exposure to high humidity. After a 2-hour exposure to ~98% humidity, Cy5 and Cy3 mean feature intensities were increased by 18.6 ± 2.9% (p < 0.00009) and 7.0 ± 2.5% (p < 0.015), respectively, over initial mean intensity levels. After a 4-hour exposure, Cy5 and Cy3 mean intensities were increased by 6.8 ± 2.2% (p < 0.002) and 5.6 ± 1.5% (p < 0.12), respectively, over initial mean intensity levels.

## Discussion

Data from microarray experiments are affected by many variables including microarray printing, RNA extraction and purification, cDNA production, dye incorporation, and hybridization conditions [7]. A breakdown in any of the critical elements of these procedures can lead to unreliable microarray data. The data presented here, and elsewhere [4], demonstrate that even at the very last step in the process of generating microarray data, i.e., the time interval between the final hybridization wash and the completion of microarray scanning, substantial errors in the Cy5/Cy3 ratios can be introduced.

A typical microarray experiment in our laboratory involves handling 5 or 6 microarrays in a batch. Considering that it takes 6 minutes to scan a 20,000 feature microarray slide, the microarrays may be exposed to ozone from 1 to 36 minutes. As illustrated in Figure 3, significant degradation of the Cy5 signal intensity occurs even at ~25 ppb ozone during this period of time, while the Cy3 signal intensity remains stable. Thus, the signal intensity ratio used for gene expression calculations changes with the amount of time the microarray is exposed to ozone. This, of course, would introduce technical variability within the microarray experiment and mask true experimental affects. In addition, there are several high throughput scanning devices that are capable of batch microarray scanning in which the microarray slides are exposed to laboratory ozone levels for prolonged and varying periods of time. These instruments can take as long as 4 hours to scan a batch of microarrays. Clearly, this would not be suitable in a laboratory without some means of dramatically reducing ozone levels.

LOWESS normalization is a method used to normalize a two-color array gene expression dataset to compensate for non-linear dye-bias [8] and we typically use this normalization method before further data analysis. To examine the consistency of the microarray data during the 8 scans shown in Figure 3A, correlations were calculated between the data obtained from each scan and the data from the initial scan, before and after LOWESS normalization. As shown in Table 1, this normalization method resulted in improvements in the correlation of the gene expression ratios in the non-carbon-filtered data set. However, this improvement does not result in the highly consistent data derived under the carbon-filtered conditions. The main reason for this is that the Cy5 feature intensities do not decay at a uniform rate, resulting in low correlations among scans done at different times (see Table 1). In other words, the data obtained from the initial scans will not be consistent with data collected at later times, and will lead to erroneous results. Because of the non-uniform reduction in Cy5 feature intensities, no standard systematic normalization can correct for data collected under high ozone conditions.

Dye swap experiments, in which a second array is hybridized with cDNAs labeled with the opposite orientation of cyanine dyes, are often used to correct for scanning- and/or labeling-related differences between Cy5 and Cy3 dyes. These sorts of experiments require both cyanine dyes to be stable. Since only the Cy3 dye is stable while the Cy5 dye is highly susceptible to ozone-induced degradation, dye swap would result in a second array with ratio measurements that also change with the amount of time spent in high ozone conditions. Thus, dye swap would provide no improvement in either the data or the conclusions drawn from the data.

The data from the humidity experiments indicate a slight increase in fluorescence signals as a result of exposure to high humidity. The extreme humidity levels were chosen to increase the chance of detecting any influence of humidity on the decay of the cyanine dyes. In a practical sense, microarray laboratories would rarely, if ever, experience humidity in the 98–99% range, so this would not adversely impact the Cy5/Cy3 ratios in normal experiments. Although extremely high humidity may modestly affect cyanine dye fluorescence intensities, the dominant environmental factor that must be controlled is ozone concentration. Uncontrolled ozone has the potential to be the largest variable in microarray experiments.

## Conclusion

Simple installation of carbon filters in the laboratory air handling system, coupled with making the laboratory air pressure positive with respect to the adjacent area, resulted in low and consistent ozone levels. Without the laboratory modifications, the intensity of the Cy5 signal dropped dramatically over a short time period (44% in 14 minutes when outdoor ozone levels were approximately 25 ppb) while the Cy3 signal intensity remained relatively constant. Such specific, rapid, and uncontrolled degradation of the Cy5 dye results in inaccurate and highly variable gene expression measurements. LOWESS normalization could not negate the effects of the degradation of the Cy5 intensity caused by ozone in the non-carbon-filtered environment. The laboratory modification dramatically increased the stability of the Cy5 dye to match the stability of the Cy3 dye. This allowed the accurate determination of gene expression by microarray using Cy5 and Cy3 fluorescent dyes.

## Methods

### Determination of HEGA filter size

Two factors are required to determine the proper size of the filter: (1) the maximum supply air flow rate and (2) the maximum pressure drop across the filter. Generally, the maximum supply air flow rate of a particular laboratory can be calculated by determining the maximum flow-rate across the fume-hood(s). For example, a laboratory with a single 5 foot fume hood and a maximum sash opening height of 2 feet will create a sash opening area of 10 ft^2^. The total flow rate is the sash opening (10 ft^2^) multiplied by the fume hood minimum face velocity (100 ft/minute is typical for chemical fume hoods) or 1,000 ft^3^/minute. To determine the maximum pressure drop across the HEGA filter, maximize the air flow rate to the laboratory by opening the fume hood sash and lowering the thermostat. If the damper is wide open, that is an indication that there is not enough static pressure in the duct to overcome the additional resistance of the HEGA filter without modifying the fan speed or increasing the size of the duct or dampers. If the damper is not fully open, there is potentially enough pressure in the duct to overcome the added resistance of a new HEGA filter. The maximum pressure available can be determined by the following process. (1) Measure the static pressure at the location of the proposed HEGA filter. (2) Add resistance to the duct at the supply air diffusers until the supply damper is fully open. (3) Measure the static pressure reading at the location of the proposed HEGA filter under these conditions. The difference between the two readings will determine the maximum pressure permitted across the new HEGA filter.

### Carbon-filtration of laboratory environment

The process for adding carbon filtration to our existing HVAC system involved several steps. Using the maximum supply air flow rate and the maximum pressure drop across the filter as described above, National Center for Toxicological Research engineers selected an appropriate carbon loaded non-woven filter. The HEGA filter series 2653 (part number 11–17979; Filtration Group, Joliet, IL, USA) which is a 24" × 24" × 12" carbon filter and which has a low pressure drop of 0.3 inches (water column) at the maximum air flow rate of 1,100 cubic feet per minute was used. Because of the low pressure drops, these gas phase carbon filters can be successfully installed in many high pressure systems provided that enough space exists above the ceiling for the filter housing.

Although the carbon filter prevented the vast majority of environmental ozone from entering the laboratory, without the laboratory air pressure being positive with respect to adjacent spaces, ozone-contaminated air may leak in around door frames and the unsealed perimeter of the laboratory. In both variable air volume (VAV) and constant air volume (CAV) HVAC systems, the exhaust box is designed to track the supply air flow with a typical adjustable offset of 50 to 100 ft^3^/minute. For example, when the exhaust VAV box tracks the supply VAV box with a positive offset of 100 ft^3^/minute and the supply is 1,000 ft^3^/minute, the exhaust will be 1,100 ft^3^/minute. The 100 ft^3^/minute offset will make the laboratory negatively pressurized with respect to its adjacent spaces and 100 ft^3^/minute of air from the surrounding areas would infiltrate the lab. The HEGA filter is effective at removing ozone to 2 ppb or less. If the ozone level in the surrounding area is 50 ppb, the laboratory would contain a mixture of 1,000 ft^3^/minute at 2 ppb ozone and 100 ft^3^/minute at 50 ppb. The overall ozone level would increase from 2 ppb to 6.4 ppb based on the following calculation: (1,000/1,100) × 2 + (100/1,100) × 50 = 6.4 ppb. To eliminate this increase in ozone and maintain the ozone level at approximately 2 ppb, the laboratory must be positively pressurized with respect to the hallway.

### In-house produced microarrays

In-house printed microarrays were constructed using the mouse 20,000 oligonucleotide set and the rat 10,000 oligonucleotide from MWG (High Point, NC, USA). The oligonucleotides were dissolved in 1× MWG Spotting Buffer A at a concentration of 20 μM and printed on poly-L-lysine-coated slides (Erie Scientific, Portsmouth, NH) using an OmniGrid Microarrayer (GeneMachines, San Carlos, CA). Printed slides were processed and stored in a desiccator at room temperature before use [9].

Target cDNAs were labeled with cyanine dyes using cDNA indirect labeling protocol [9]. Cy3- and Cy5- labeled cDNAs were mixed together and concentrated to a volume of less than 5 μl using a SpeedVac SPD 1010 (Themo Savant, Holbrook, NY) at room temperature. The samples were then mixed with 60 μl of pre-warmed hybridization buffer. Detailed hybridization and washing procedures have been described previously [9].

### Commercial microarrays

Microarrays (22K mouse oligonucleotides) manufactured by Agilent Technologies (Cat. No. G4121A) were hybridized according to Agilent protocols. Total RNAs were labeled using the Agilent Low RNA Input Fluorescent Linear Amplification Kit (Cat. No. 5184-3523). The hybridization and washing procedures were also performed following the Agilent 60-mer Oligo Microarray Processing Procedure (Cat. No. G4140-90030).

### Scanning, feature extraction, and data analysis

Immediately after the slide washing and spin-drying procedures, the microarrays were scanned using the Axon 4000B microarray scanner (Molecular Devices, Sunnyvale, CA, USA) with photomultiplier tube settings balanced for the 635 nm and 532 nm channels. Following the initial scans of paired microarrays in the carbon-filtered laboratory, each slide was placed in a separate black plastic 25-slide box with the lid ajar to permit free air flow and to block direct light from striking the microarray. One box was placed in the adjacent hallway in which the air is not carbon-filtered; the other box remained in the lab with the carbon filtered air. The hallway was used so that ozone measurements could be made in real-time in both the laboratory (reduced ozone) and the hallway (ambient ozone). Scans of each slide of a pair were alternated for the duration of the experiment. It should be noted that the slides placed in the hallway were only exposed to atmospheric ozone when placed in the hallway and that during scanning, by necessity, they were in the carbon-filtered (reduces ozone) environment. Thus the actual ozone exposure for these slides is approximately 1/2 of the total experiment time. Ozone levels were measured using a Model 450 Ozone Monitor (Advanced Pollution Instrumentation, Inc., San Diego, CA, USA).

The resulting images were analyzed by measuring the fluorescence of all features on the microarrays using the GenePix Pro 6.0 image analysis software (Molecular Devices, Sunnyvale, CA, USA). The median fluorescence intensity of all the pixels within one feature was taken as the intensity value for that feature. All the raw data were imported into ArrayTrack Software [10] and were normalized using LOWESS Normalization with background subtraction. The data correlation values were computed with JMP 6.0 software (SAS, Inc., Cary, NC).

## Competing interests

The author(s) declare that they have no competing interests.

## Authors' contributions

WSB, TH, VGD, and JCF participated in the design of experiments. WSB, TH, and CDM carried out the experiments. CLM prepared all samples. WSB, TH, and JCF analyzed the data. ATS designed and implemented the laboratory ozone filtration system. WSB and JCF drafted the manuscript. All authors approved the final manuscript.

## Figures and Tables

**Table 1:** Effects of LOWESS Normalization on Correlations.

Effects of LOWESS Normalization									
	Scan Interval (minutes):	0	13	26	39	52	65	78	91

Non- carbon-filtered	Raw ratio data	1.00	0.93	0.81	0.69	0.59	0.48	0.40	0.37
	Normalized ratio data	1.00	0.98	0.96	0.94	0.92	0.88	0.84	0.80
	Cy5 Intensity data	1.00	0.98	0.96	0.94	0.90	0.85	0.80	0.76
	Cy3 Intensity data	1.00	0.99	0.99	0.99	0.98	0.98	0.98	0.97

Carbon-filtered	Raw ratio data	1.00	0.99	0.99	0.99	0.98	0.98	0.97	0.96
	Normalized ratio data	1.00	0.99	0.99	0.99	0.98	0.98	0.98	0.98
	Cy5 Intensity data	1.00	0.99	0.99	0.99	0.99	0.99	0.99	0.99
	Cy3 Intensity data	1.00	0.99	0.99	0.99	0.99	0.99	0.99	0.99

Intensity data and Cy5/Cy3 ratio (raw) data were obtained for individual microarray features at each scan time using the Axon GenePix Pro software. LOWESS normalization was performed on the ratio data. The data shown are correlations (r-values) of the raw or normalized data at each scan interval compared to the 0-minute scan interval.
